# Validation of a criteria-specific long-term survival prediction model for hepatocellular carcinoma patients after liver transplantation

**DOI:** 10.1038/srep11733

**Published:** 2015-06-22

**Authors:** Fei Teng, Qiu-Cheng Han, Guo-Shan Ding, Zhi-Jia Ni, Hong Fu, Wen-Yuan Guo, Xiao-Min Shi, Xiao-Gang Gao, Jun Ma, Zhi-Ren Fu

**Affiliations:** 1 Organ Transplantation Institute of Changzheng Hospital, Second Military Medical University, Shanghai 200003, China

## Abstract

The aim of this study was to validate a criteria-specific long-term survival prediction model (MHCAT) in a large cohort of hepatocellular carcinoma (HCC) patients after liver transplantation (LT) in China. Independent risk factors in MHCAT were retrospectively analysed for HCC patients recorded in the China Liver Transplant Registry. Survival predictions for each patient were calculated using MHCAT scores and the Metroticket formula separately, and the prediction efficacy of MHCAT and Metroticket was compared using the area under ROC curve (*c*-statistic). A total of 1371 LTs for HCC were analysed in the study, with a median follow-up of 22.2 months (IQR 6.1–72.4 months). The proportions meeting the Milan, UCSF, Fudan and Hangzhou criteria were 34.4%, 39.7%, 44.2% and 51.9%, respectively. The *c*-statistics for MHCAT predictions of 3- and 5-year survival rates of HCC recipients were 0.712–0.727 and 0.726–0.741, respectively. Among these patients, 1298 LTs for HCC were ultimately selected for the comparison analysis for prediction efficacy. The *c*-statistic of MHCAT for predictions of 3-year survival with reference to the Milan, UCSF and Fudan criteria was significantly increased compared with that for Metroticket (p < 0.05). In conclusion, MHCAT can effectively predict long-term survival for HCC recipients after LT.

Hepatocellular carcinoma (HCC) is the fifth most frequent and the third most deadly cancer in the world^1^. Over the past 15 years, the occurrence of HCC has more than doubled. Each year 500,000 new cases and 360,000 deaths are reported in the Asia-Pacific region, over 60% of which occur in China^2^. Since liver transplantation (LT) was first utilized, it was widely recognized as an effective treatment for HCC given that it could cure both the tumour and underlying liver diseases. However, access to transplantation is a balance between maximizing a patient’s chances of cure and overall survival due to the scarcity of liver donations. Criteria for LT and organ allocation systems have led to various controversies over LT for HCC patients in terms of whether and to what extent the criteria should be less restrictive. A number of extended criteria were proposed based on the Milan criteria (a solitary HCC nodule 5.0 cm or less in diameter or no more than three tumour nodules with the largest lesion 3.0 cm or less in diameter without tumour invasion of blood vessels or lymph nodes), such as the University of California San Francisco (UCSF) criteria (similar to the Milan criteria, extending the diameter to 6.5 cm for a solitary nodule, 4.5 cm for the largest nodule, and 8.0 cm as the total diameter when multiple nodules are present) and Up-to-Seven criteria^3^,^4^,^5^,^6^,^7^. Transplant scientists in China have also established the Shanghai Fudan criteria (similar to the UCSF criteria, extending the diameter to 9.0 cm for a solitary nodule, 5.0 cm for the largest nodule, and 9.0 cm as the total diameter when multiple nodules are present) and Hangzhou criteria (a total tumour diameter 8 cm or less or a total tumour diameter of greater than 8 cm with an Edmondson grade I or II and pre-operative alpha-fetoprotein level of 400 ng/mL or less simultaneously).

Carefully considering peri-operative risk factors, we developed a criteria-specific long-term survival prediction model for HCC patients after LT (MHCAT) based on the Milan, UCSF, Fudan and Hangzhou criteria^8^. Our objectives in this study were to compare the efficacy of survival prediction between the MHCAT and Metroticket systems and to validate MHCAT in LT for Chinese HCC patients using the large cohort of HCC cases with adequate follow-up recorded in the China Liver Transplant Registry (CLTR).

## Results

### Patient Characteristics

All LTs were ABO-type compatible. Among the 1371 patients, 1244 (90.8%) were male, and 126 (9.2%) were female; the mean age was 49.8 ± 9.1 years. A total of 1309 (95.5%) patients received a LT from a deceased donor, whereas 62 (4.5%) patients received a LT from a living donor. The median follow-up time was 22.2 months (IQR, 6.1–72.4 months). The 1-, 3-, and 5-year cumulative survival rates were 63.1%, 43.8%, and 37.6%, respectively, with a total number of 748 deaths from any cause, and tumour recurrence was noted in 247 patients in total. The 1-, 3- and 5-year HCC recurrence-free survival rates after LT were 55.8%, 40.4% and 35.7%, respectively. Table 1 presents patient characteristics regarding MHCAT. Compared with the Milan criteria, the UCSF, Fudan and Hangzhou criteria expanded the population meeting the criteria by 15.53%, 28.51% and 51.28%, respectively. Population expansions among different criteria are presented in Supplement 1, 2, and 3.

### Validation of MHCAT in patients with HCC after LT in China

**Figure 1:**
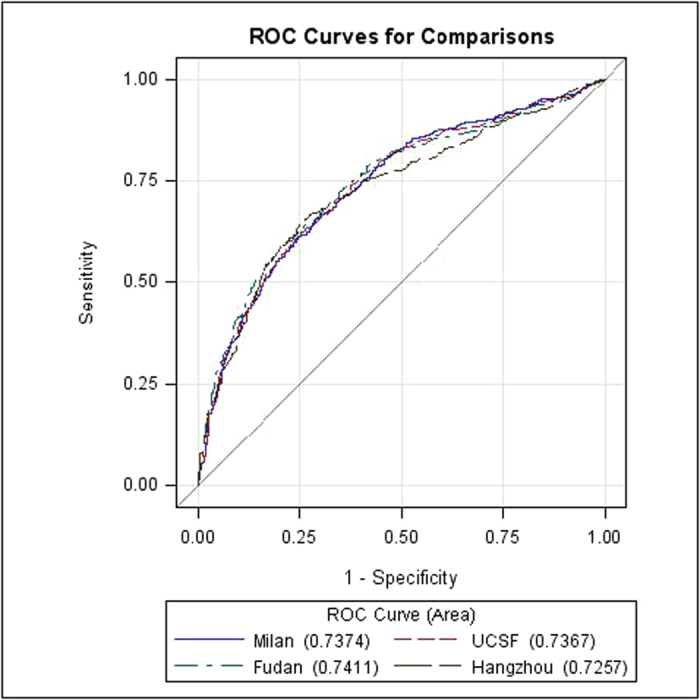
ROC curve for MHCAT predicting 5-year survival after LT.

Each of the 1371 patients was evaluated using MHCAT with reference to the Milan, UCSF, Fudan, and Hangzhou criteria, and the individual 3- and 5-year predicted survival rates were calculated using MHCAT scores. By comparing the predicted rates with the actual rates, ROC curves were generated for the 3- and 5-year survival predictions of MHCAT system (Supplement 4 and Fig. 1). The areas under the ROC curves were 0.712–0.727 for 3-year survival predictions and 0.726–0.741 for 5-year survival predictions. The high area under the ROC values, of which no significant differences were noted using the Kruskal-Wallis tests (*p* > 0.05), indicated that MHCAT exhibited a good performance in predicting the long-term post-transplant survival of HCC patients.

**Figure 2:**
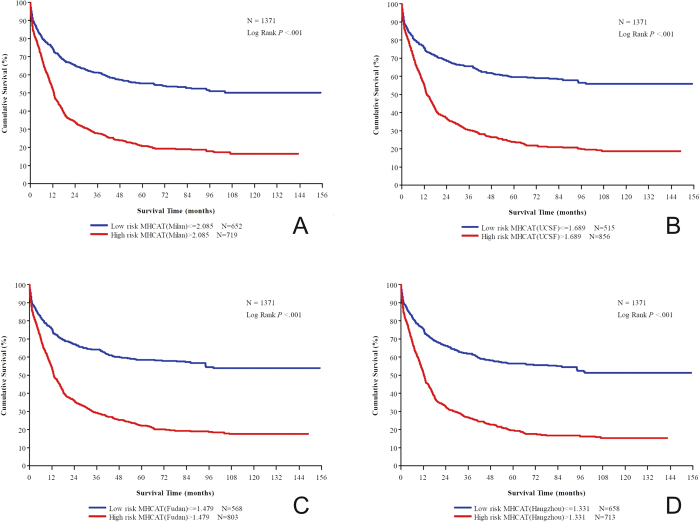
Overall survival analysis based on cut-off values for the MHCAT scoring system (p < 0.001 for all). (**A**) Milan criteria; (**B**) University of California San Francisco criteria; (**C**) Shanghai Fudan criteria; (**D**) Hangzhou criteria.

The primary endpoint of our study on MHCAT was the survival status at five years after liver transplantation. In addition, the area under the ROC curve for 5-year survival was considerably increased compared with the 3-year survival curve. Therefore, the best MHCAT score cut-off values were obtained from 5-year survival ROC curves. The best MHCAT score cut-off values to stratify low and high risk for post-transplant mortality were 2.085, 1.689, 1.479, and 1.331 for the Milan, UCSF, Fudan, and Hangzhou criteria, respectively. Regardless of the criteria adopted, Kaplan–Meier analysis revealed significantly increased overall survival and recurrence-free survival in low-risk patients compared with high-risk patients (Fig. 2 and Supplement 5, *p* < 0.001). Supplement 6 and 7 present the overall survival and recurrence-free survival for 1, 3, and 5 years in different subgroups.

### MHCAT and Metroticket comparisons

**Figure 3:**
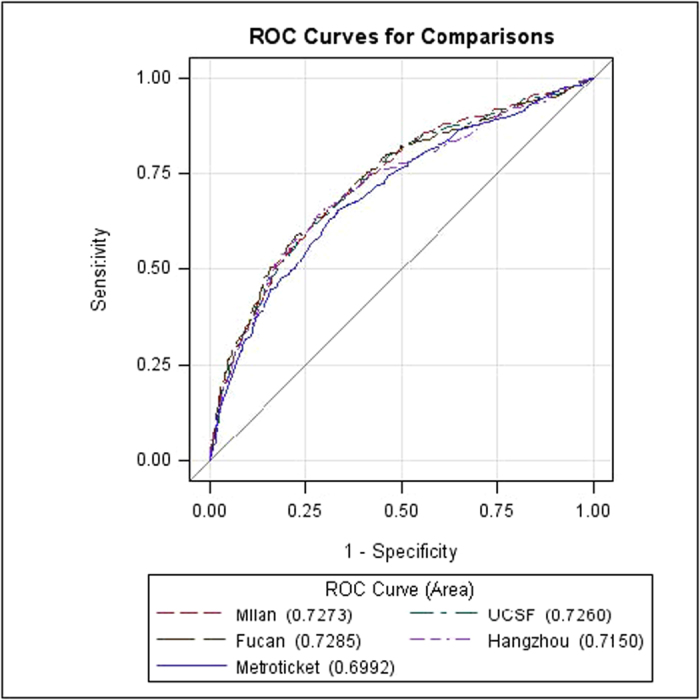
Comparison between MHCAT and Metroticket 3-year survival predictions.

**Figure 4:**
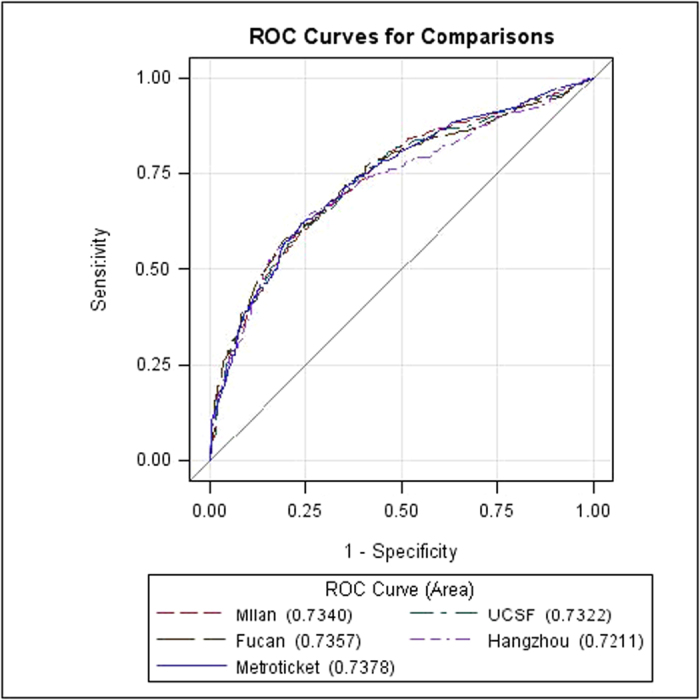
Comparison between MHCAT and Metroticket 5-year survival predictions.

According to the Metroticket formula, a predicted 3-year and 5-year survival of each HCC recipient can be calculated based on the known number of tumours, size of the largest tumour and vascular invasion. Among the 1371 patients, 73 patients had missing or abnormal data on the number of tumours or the largest tumour size. Therefore, 1298 patients were ultimately selected for the MHCAT and Metroticket comparative analysis. ROC curves were generated for the three- and five-year survival predictions of MHCAT and the Metroticket system (Figs 3 and 4). The *c*-statistics of the Metroticket system for 3- and 5-year survival predictions were 0.699 and 0.738, respectively. Kruskal-Wallis analysis showed that the *c*-statistic of MHCAT based on the Milan, UCSF and Fudan criteria was significantly increased compared with the Metroticket 3-year survival prediction (p < 0.05), whereas no differences in 5-year survival predictions were noted between Metroticket and MHCAT, regardless of any one of the four criteria (p > 0.05). We measured the number of true 3-year and 5-year survival predictions using different MHCAT with reference to the four criteria as well as Metroticket system. Compared with the Metroticket system, the accuracy of predicting patients the number of patients dead at 3 or 5 years using MHCAT was considerably higher, whereas the accuracy of predicting the number of living patients was lower. Furthermore, the overall accuracy of predicting survival status using MHCAT was substantially increased (Tables 2 and 3).

## Discussion

With over 26,000 LT cases, China ranks second only to the USA. However, the five-year survival after LT in China is significantly reduced compared with the USA and Europe (60.5% *vs*. 73.7% and 73.0%, respectively)^9^,^10^,^11^. However, the survival gap of patients with benign end-stage liver diseases is small: 73.2% in China, 74.1% in America and 73.2% in Europe. A low curative effect is mainly responsible for the low survival rate of HCC patients in China compared with those in the USA and Europe (49.7% *vs*. 67.5% and 64.0%, respectively). HCC accounts for 16% and 20.9% of all LT cases in Europe and the USA, respectively^9^,^11^. In China, the rate is as high as 40%^10^. Moreover, 65.6% of HCC patients in China exceeded the Milan criteria before transplantation, which inevitably affected their long-term survival. The MHCAT was built with reference to the four most representative HCC LT criteria using accurate HCC patient data from a single centre in China with a follow-up of at least six years. By validating its prediction efficacy in a large cohort of HCC patients in China, this model may help clinicians determine what type of HCC patient should receive LT as well as determine individual therapies administered to patients.

HCC patients meeting the Milan criteria have achieved satisfactory 5-year post-transplant survival over the past two decades of liver transplantation, which is similar to that observed for benign liver diseases. A number of other extended criteria were proposed to explore the ceiling of tumour load with acceptable long-term survival. However, controversies arose concerning the criteria adopted as prioritization tools for organ allocation or transplant programs. To some HCC patients, the yes-no question might indicate the chance to receive a liver transplantation and survive. In this sense, MHCAT might be a more rational and equitable quantification tool.

Increasing evidence demonstrates that additional risks, such as AFP, microvascular invasion, or response to TACE, independently affect post-transplant survival or HCC recurrence in addition to conventionally accepted factors, such as tumour size, number and major vascular invasion^12^,^13^,^14^. Among the independent predictors of MHCAT, the criteria for LT primarily represent tumour morphological characteristics, whereas AFP reflects the tumour biological activity at some level. In this post-transplant survival prediction model, we included intraoperative blood loss for the first time because circulating tumour cells likely plat a significant role in HCC recurrence. Based on all of these risk factors, MHCAT demonstrated increased efficacy compared with Metroticket for 3-year survival predictions and a comparable 5-year survival prediction efficacy.

Although other criteria have demonstrated satisfactory performances, the Milan criteria, which was integrated into the Union for International Cancer Control (UICC) TMN and United Network for Organ Sharing (UNOS) staging systems for HCC, continues to play a leading role among all types of criteria. Patients meeting the Milan criteria receive extra MELD scores in the current organ allocation system; thus, it is inappropriate to replace the Milan criteria with other criteria. However, we can provide HCC patients a MHCAT score on the basis of the Milan criteria in combination with other predictors. Thus, the coefficients for the Milan criteria and other predictors were established, which helped us to optimize the allocation system using a modified extra MELD score.

In addition to the increased prediction efficacy, the most important advantage for MHCAT is the capacity to process the unavoidable discrepancies between pre-transplant imaging interpretation and ex-plant pathology examination. When the decision for LT is made for an HCC patient, predictions of size and the number of nodules are based upon imaging. Pre-transplant staging fails to predict the number of hepatocellular tumours at pathology in approximately 25 to 35% of patients^15^,^16^,^17^. Approximately 28% of patients within the Milan criteria were underestimated by pre-transplant imaging^15^. Our study indicates that the use of the UCSF, Fudan, and Hangzhou criteria resulted in an obvious expansion of the number of eligible patients by 15.53%, 11.65%, and 19.66%, respectively, compared with the Milan criteria, whereas the efficacy of MHCAT with reference to the four different criteria remained fairly consistent. This consistency suggests that we can use the next expanded criteria for MHCAT scoring when an HCC patient demonstrates a borderline score on one criterion.

The major limitation of the current model is that intraoperative blood loss cannot be predicted before transplantation, hence weakening the role of MHCAT on HCC candidate selection. It is generally agreed that a rational approach that considers markers of tumour behaviour might be useful to select HCC patients for transplantation. Yet, no potential biological, histologic, or molecular markers have been implemented to date in routine practice for selecting HCC candidates. As liver transplantation completely removes primary tumours and potential intrahepatic metastases, HCC recurrence is largely due to circulating tumour cells (CTCs) that existed prior to transplantation^18^. Intraoperative blood loss may facilitate tumour cell entry into the circulation. Therefore, the counting and culturing of CTCs acts as a promising substitution for intraoperative blood loss or possibly a more valuable predictor^1^,^19^,^20^.

A second potential limitation of our study is that the 5-year survival was approximately 54.1 to 59.0% even in low-risk groups. Given that only 34.4% of HCC patients met the Milan criteria in our cohort, the overall long-term survival was quite low. In future studies, we should determine cut-off values that distinguish HCC subgroups with a 5-year survival of greater than 65%, which is comparable with that observed in patients with benign liver diseases.

In conclusion, we validated the reliability of MHCAT in long-term survival predictions using a large cohort of HCC patients after liver transplantation. Compared with Metroticket, MHCAT exhibited enhanced prediction efficacy for 3-year survival and similar efficacy for 5-year survival, thus making MHCAT a useful tool for selecting HCC transplant candidates.

## Methods

### Patients

**Figure 5:**
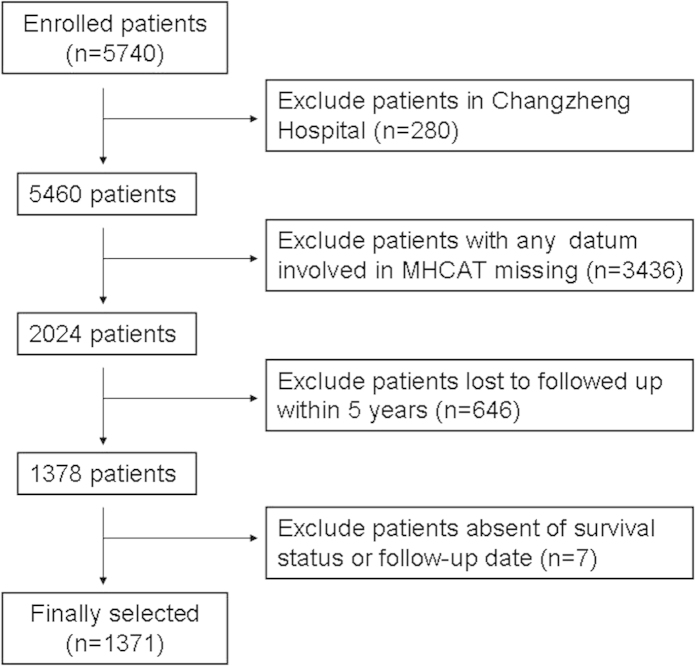
An illustration of the patient selection process.

The study population consisted of 5740 adult patients with HCC who underwent transplantation in 81 centres in China from January 1980 to June 2008. The inclusion criteria were as follows: (1) patients older than 18 years of age, and (2) histopathological proof of HCC on the explanted liver. The exclusion criteria were as follows: (1) combined transplantation, (2) incidental HCC, and (3) mixed carcinoma and other malignant hepatic tumours. Among the initial 5740 patients, 280 (4.9%) patients for whom LTs were performed at Changzheng Hospital, Shanghai were excluded as the MHCAT was constructed using this cohort of single-centre patients. In addition 3436 (59.9%) patients were excluded because at least one datum involved in MHCAT (four criteria for HCC, pre-transplant AFP and total bilirubin value, re-transplantation, and intraoperative blood loss) was missing; the individual MHCAT score for each HCC patient enrolled cannot be obtained if any datum involved in MHCAT was missing. Third, 646 (11.3%) patients were excluded because they were lost to follow-up within 5 years. Fourth, 7 (0.1%) patients were excluded because their survival status or follow-up dates were missing. Finally, the MHCAT was used for long-term survival predictions in a cohort of 1371 (23.9%) patients with HCC after LT in China. Figure 5 illustrates the patient selection process.

### Data collection

In May 2008, the CLTR was authorized by the Ministry of Health of the People’s Republic of China as the national registry system for liver transplantation. Pre-transplant data at enrolment and post-transplant events were collected retrospectively by independent clinical research assistants in each liver transplantation centre and entered into the CLTR database. Data on the main tumour size, number of tumours, vascular invasion, and conditions of satisfying or dissatisfying different criteria were collected based on the final pathology review of the explanted liver. The study proposal, which was consistent with the ethical guidelines of the 1975 Declaration of Helsinki, was presented to CLTR and approved by Scientific Committee of CLTR in 2013. According to CLTR policy, researchers cannot obtain original data, and the statistical analysis is independently conducted by the CLTR system and statisticians complying with the study proposal. All methods were performed in accordance with the approved guidelines for CLTR clinical studies.

### MHCAT and Metroticket

MHCAT with reference to the Milan, UCSF, Shanghai Fudan, and Hangzhou criteria have been reported elsewhere. Supplement 8 shows the prediction formula for 3-year and 5-year survival of HCC patients after LT according to MHCAT score. The Metroticket Calculator is available at http://www.hcc-olt-metroticket.org/calculator/.

### Statistical Analysis

Statistical analyses were conducted independently by a professional statistician of CLTR using SAS V9.2 (SAS institute, Cary, NC, USA) and checked by another senior statistician. Continuous variables were reported as the mean ± SD or as median (q1, q3) if the variable was not normally distributed. Categorical variables were reported as frequencies (%). The primary outcome was death or re-transplantation of patients. Time was censored at the date of last follow-up assessment for patients who were still alive. The receiver operating characteristic (ROC) curve was used to determine the efficacy of MHCAT and Metroticket, and the area under the ROC curve (*c*-statistic) was compared using Kruskal-Wallis analysis. To differentiate two groups of patients with significantly different risks of mortality, a cut-off value was derived from the area under the ROC curve of the score based on the highest Youden index. The Kaplan-Meier method with the log-rank test was used to analyse long-term survival rates. P-values were estimated in a two-tailed manner. Differences were considered statistically significant at p < 0.05.

## Additional Information

**How to cite this article**: Teng, F. *et al.* Validation of a criteria-specific long-term survival prediction model for hepatocellular carcinoma patients after liver transplantation. *Sci. Rep.* **5**, 11733; doi: 10.1038/srep11733 (2015).

## Supplementary Material

Supplementary Information

## Figures and Tables

**Table 1:** Characteristics of hepatocellular carcinoma patients based on the MHCAT.

Variables		Value
Meeting Milan Criteria		472 (34.4%)
Meeting UCSF Criteria		544 (39.7%)
Meeting Shanghai Fudan Criteria		606 (44.2%)
Meeting Hangzhou Criteria		712 (51.9%)
Re-transplantation		49 (3.6%)
AFP (ng/ml)	mean ± SD	12828.0 ± 249523.4
	median (q1, q3)	148.1 (15.0, 1235.0)
TB (ng/ml)	mean ± SD	60.6 ± 89.1
	median (q1, q3)	30.9 (18.3, 58.9)
Intraoperative blood loss (IU)	mean ± SD	12.4 ± 15.7
	median (q1, q3)	7.5 (4.0, 15.0)

TB, total bilirubin; AFP, alpha-fetoprotein; UCSF, University of California San Francisco.

**Table 2:** True 3-year survival predictions using different MHCAT systems based on the four criteria and the Metroticket system.

Survival status at 3 years		Alive (n)	Dead (n)	Accuracy (%)
Actual		582	716	–
Predicted	MHCAT (Milan)	363	416	60.0%
	MHCAT (UCSF)	369	415	60.4%
	MHCAT (Fudan)	359	423	60.2%
	MHCAT (Hangzhou)	344	435	60.0%
	Metroticket	414	255	51.5%

**Table 3:** True 5-year survival status predictions using different MHCAT systems with reference to the four criteria and the Metroticket system.

Survival status at 5 years		Alive (n)	Dead (n)	Accuracy (%)
Actual		502	796	–
Predicted	MHCAT (Milan)	363	416	61.7%
	MHCAT (UCSF)	369	415	61.9%
	MHCAT (Fudan)	359	423	61.9%
	MHCAT (Hangzhou)	344	435	61.8%
	Metroticket	414	255	53.8%
