# Supplementary material for: Validation of a criteria-specific long-term survival prediction model for hepatocellular carcinoma patients after liver transplantation
Source: Sci Rep. 2015 Jun 22;5:11733. doi: 10.1038/srep11733 (PMC4476095; doi:10.1038/srep11733)
Supplement: Supplementary Information [file srep11733-s1.pdf]

# Validation of a criteria-specific long-term survival prediction model for hepatocellular carcinoma patients after liver transplantation

**Fei Teng[1*], Qiu-Cheng Han[1*], Guo-Shan Ding[1], Zhi-Jia Ni[1], Hong Fu[1], Wen-Yuan Guo[1], Xiao-Min Shi[1], Xiao-Gang Gao[1], Jun Ma[1], Zhi-Ren Fu[1△]**

Supplement 1. Population expansion of the UCSF, Fudan and Hangzhou criteria compared with the Milan criteria.

| | Meeting the Milan* | Meeting the UCSF but exceeding the Milan | Meeting the Fudan but exceeding the Milan | Meeting the Hangzhou but exceeding the Milan |
|---|---|---|---|---|
| N | 470 | 73 | 134 | 241 |
| PE**（%） | -- | 15.53 | 28.51 | 51.28 |

* excluding patients meeting the Milan but exceeding the UCSF or Hangzhou criteria (n=2).

** PE, population expansion.

Supplement 2. Population expansion of the Fudan and Hangzhou criteria compared with the UCSF criteria.

| | Meeting the UCSF* | Meeting the Fudan but exceeding the UCSF | Meeting the Hangzhou but exceeding the UCSF |
|---|---|---|---|
| N | 541 | 63 | 171 |
| PE**（%） | -- | 11.65 | 31.61 |

* excluding patients meeting the UCSF but exceeding the Hangzhou criteria (n=3).

** PE, population expansion.

Supplement 3. Population expansion of the Hangzhou criteria compared with the Fudan criteria.

| | Meeting the Fudan* | Meeting the Hangzhou but exceeding the Fudan |
|---|---|---|
| N | 595 | 117 |
| PE**（%） | -- | 19.66 |

* excluding patients meeting the Fudan but exceeding the Hangzhou criteria (n=11).

** PE, population expansion.

Supplement 4. ROC curve for MHCAT predicting 3-year survival after LT.

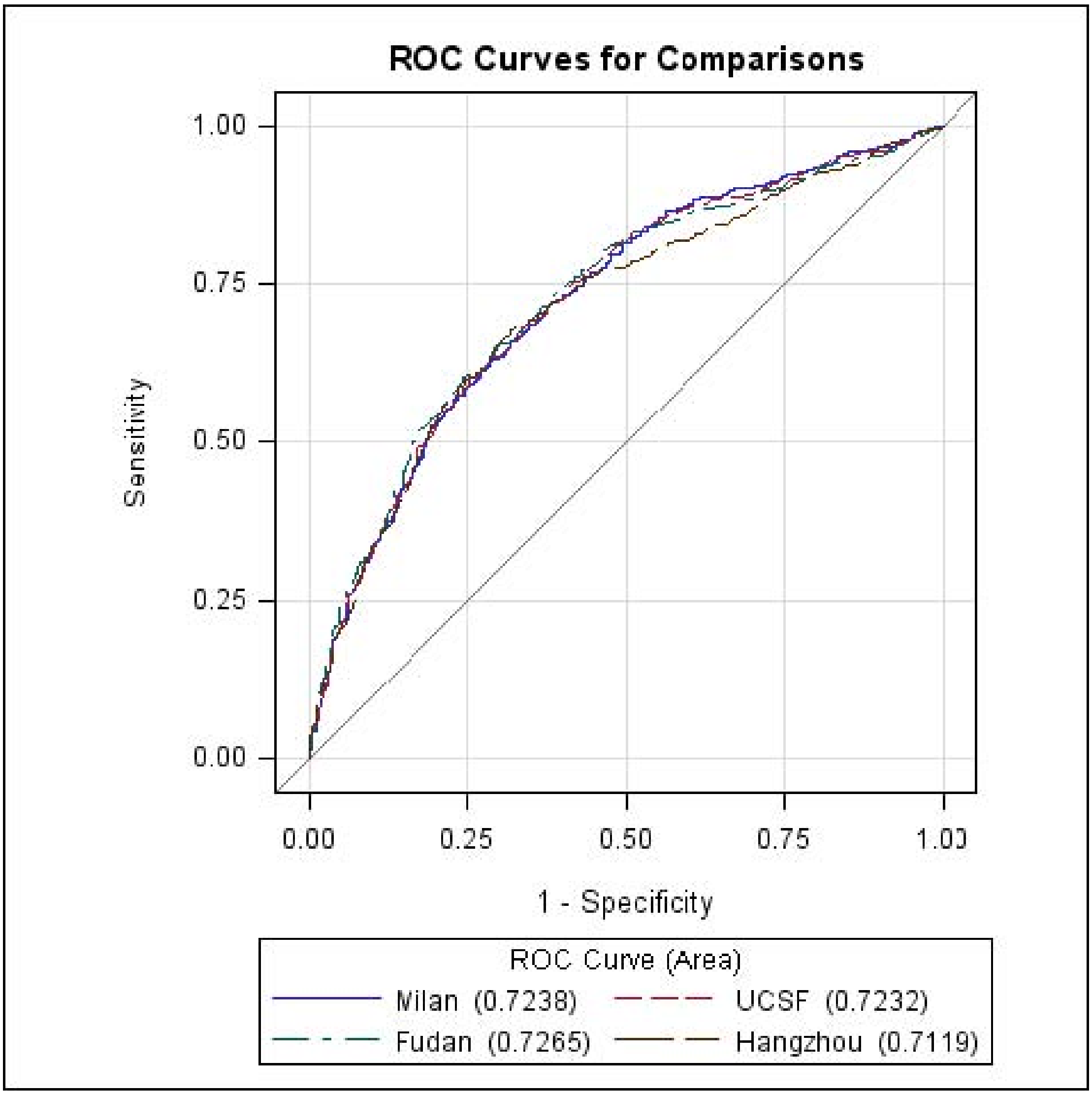

Supplement 5. Recurrence-free survival analysis based on cut-off values for the MHCAT scoring system ($p < 0.001$ for all). (A) Milan criteria; (B) University of California San Francisco criteria; (C) Shanghai Fudan criteria; (D) Hangzhou criteria.

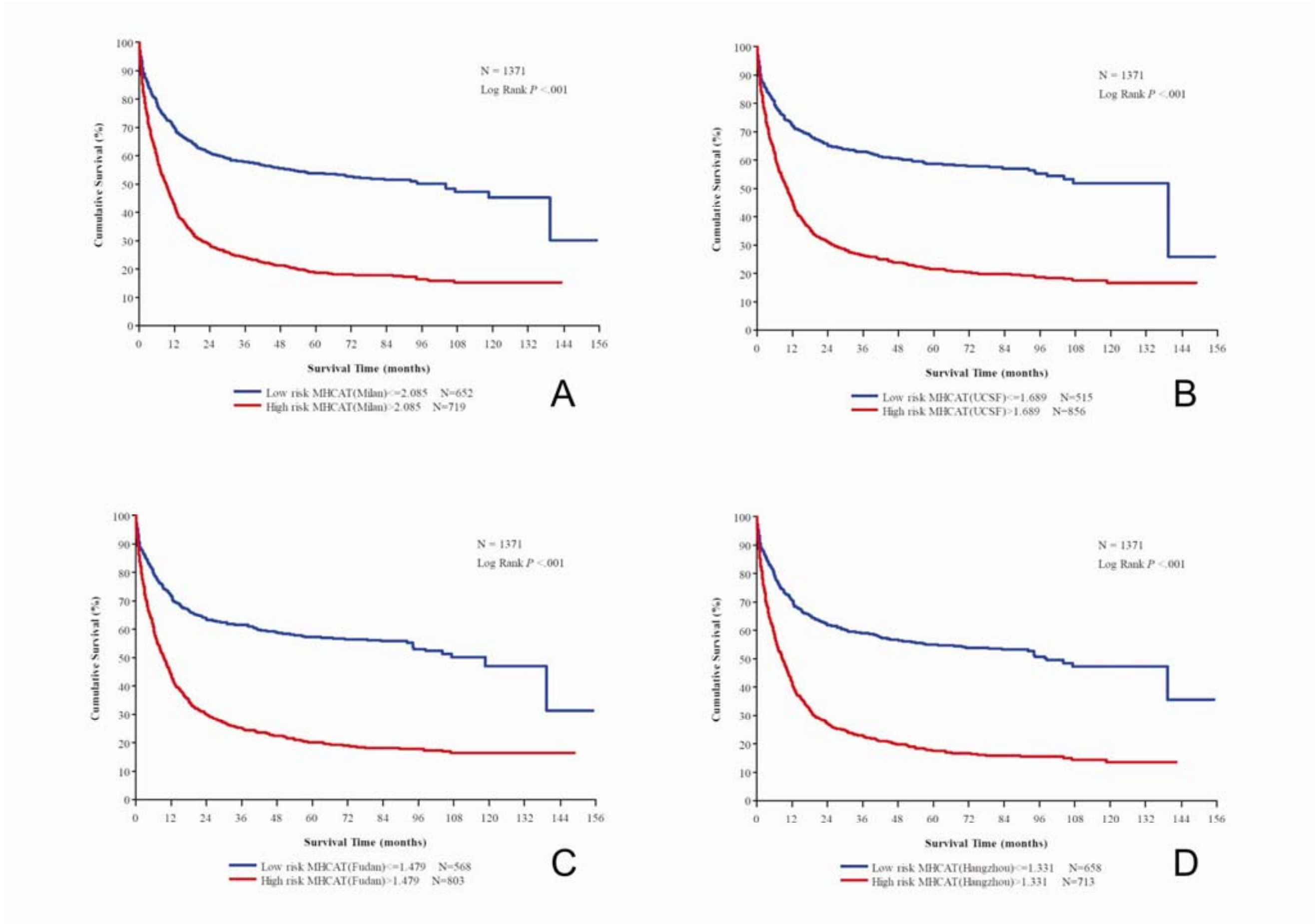

Supplement 6. The overall survival for HCC patients with low risk and high risk based on MHCAT scoring system.

| Criteria | MHCAT score | N | Overall survival rate (%) | | | p value |
|---|---|---|---|---|---|---|
| | | | 1-year | 3-year | 5-year | |
| Milan | ≤2.085 | 652 | 74.9 | 61.4 | 55.5 | ＜0.001 |
| | ＞2.085 | 719 | 52.4 | 28.0 | 20.9 | |
| UCSF | ≤1.689 | 515 | 75.9 | 65.8 | 59.8 | ＜0.001 |
| | ＞1.689 | 856 | 55.4 | 30.6 | 23.8 | |
| Fudan0 | ≤1.479 | 568 | 75.7 | 64.3 | 58.6 | ＜0.001 |
| | ＞1.479 | 803 | 54.2 | 29.4 | 22.3 | |
| Hangzhou | ≤1.331 | 658 | 75.7 | 62.2 | 56.5 | ＜0.01 |
| | ＞1.331 | 713 | 51.5 | 26.9 | 19.6 | |

Supplement 7. The recurrence-free survival for HCC patients with low risk and high risk based on MHCAT scoring system.

| Criteria | MHCAT score | N | Recurrence free survival rate (%) | | | p value |
|---|---|---|---|---|---|---|
| | | | 1-year | 3-year | 5-year | |
| Milan | ≤2.085 | 652 | 70.1 | 58.1 | 54.1 | <0.001 |
| | >2.085 | 719 | 52.4 | 28.0 | 20.9 | |
| UCSF | ≤1.689 | 515 | 72.4 | 63.3 | 59.0 | <0.001 |
| | >1.689 | 856 | 45.8 | 26.6 | 21.6 | |
| Fudan | ≤1.479 | 568 | 72.0 | 61.8 | 57.6 | <0.001 |
| | >1.479 | 803 | 44.3 | 25.3 | 20.2 | |
| Hangzhou | ≤1.331 | 658 | 70.8 | 59.3 | 55.2 | <0.001 |
| | >1.331 | 713 | 41.9 | 23.0 | 17.7 | |

Supplement 8

$$MHCAT(Milan) = 0.093*LnAFP + 0.038*IBL + 1.429*Re + 1.504*Cri$$

$$S(3\text{-year})=0.911^{\exp\{MHCAT(Milan)\}}$$

$$S(5\text{-year})=0.888^{\exp\{MHCAT(Milan)\}}$$

$$MHCAT(UCSF) = 0.090*LnAFP + 0.038*IBL + 1.373*Re + 1.555*Cri$$

$$S(3\text{-year})=0.907^{\exp\{MHCAT(UCSF)\}}$$

$$S(5\text{-year})=0.883^{\exp\{MHCAT(UCSF)\}}$$

$$MHCAT(Fudan) = 0.091*LnAFP + 0.034*IBL + 1.296*Re + 1.361*Cri$$

$$S(3\text{-year})=0.877^{\exp\{MHCAT(Fudan)\}}$$

$$S(5\text{-year})=0.846^{\exp\{MHCAT(Fudan)\}}$$

$$MHCAT(Hangzhou) = 0.186*LnTB + 0.020*IBL + 1.312*Re + 1.520*Cri$$

$$S(3\text{-year})=0.860^{\exp\{MHCAT(Hangzhou)\}}$$

$$S(5\text{-year})=0.824^{\exp\{MHCAT(Hangzhou)\}}$$

(IBL , Intraoperative blood loss (IU); Re = 0 without retransplantation, Re = 1 with retransplantation; Cri = 0 within criteria, Cri = 1 exceeding criteria; S, probability of surviving at 3-year or 5-year after LT)
